# Understanding population structure and historical demography of *Litsea auriculata* (Lauraceae), an endangered species in east China

**DOI:** 10.1038/s41598-017-16917-x

**Published:** 2017-12-11

**Authors:** Qifang Geng, Lin Sun, Peihua Zhang, Zhongsheng Wang, Yingxiong Qiu, Hong Liu, Chunlan Lian

**Affiliations:** 1 School of Life Sciences, Nanjing University, 163 Xianlin Road, Nanjing, 210023 Jiangsu Province China; 2 State Key Laboratory of Pharmaceutical Biotechnology, School of Life Sciences, Nanjing University, 163 Xianlin Road, Nanjing, 210023 Jiangsu Province China; 3 Key laboratory of Conservation Biology for Endangered Wildlife of the Ministry of Education, and college of Life Sciences, Zhejiang University, Hangzhou, 310058 Zhejiang Province China; 4 Department of Earth and Environment, International Center for Tropical Botany, Florida International University, Miami, FL 33199 USA; 5 Asian Natural Environmental Science Center, The University of Tokyo, 1-1-8 Midori-cho, Nishitokyo, Tokyo, 188-0002 Japan

## Abstract

Detecting how historical and contemporary factors contribute to genetic divergence and genetic structure is a central question in ecology and evolution. We examine this question by intergrating population genetics with ecological niche modelling of *Litsea auriculata* (Lauraceae), which is endangered and native to east China. Geographical and environmental factors including climatic fluctuations since the last glacial maximum (LGM) have also contribute to population demography and patterns of genetic structure. *L*. *auriculata* populations underwent expansion after divergence and dramatically decreased to the current small size with relative population bottlenecks due to climate changes. Populations separated by physical geographical barrier including geographic distance and Yangtze River, as a result contemporary gene flow among *L*. *auriculata* populations showed drastic declines in comparison with historical gene flow, resulting in a high level of population divergence. Thus, patterns of genetic structure of *L*. *auriculata* can result from both geographic and environmental factors including climate changes. This information is helpful in forming conservation strategies for *L*. *auriculata* in China.

## Introduction

*Litsea auriculata* (Lauraceae) is restricted to eastern China, occurring within a limited range from 30°21′–33°33′N and 112°1′–119°26′E. The current populations of *L*. *auriculata* are only scattered and distributed in a few mountain locations, mainly due to habitat loss^1^. This species is listed as endangered in the Chinese Plant Red Book^1^. Most extant populations are small and consist of less than 40 individuals. Previous studies of *L*. *auriculata* focused on its habitat characteristics^2^ and seedling biology^3,4^, but nothing is known about its population genetics. Determining effective conservation actions for this endemic species often requires knowledge of contemporary population structure, along with an understanding of the historical processes by which populations were shaped. Although its historical geographic distribution is unknown, extant Eastern populations are thought to represent a remnant of a formerly more widespread distribution. Therefore, these geographic discrete populations of *L*. *auriculata* represent an ideal system to elucidate the roles of historical vs. contemporary processes in determining the current patterns of disconnected population remnants and genetic variation^5^.

Pleistocene climatic cycling is one of the most important drivers of contemporary diversity and genetic structure in many temperate species and communities^6^. The distributions of various organisms experienced concomitant expansions and contractions caused by the global cyclical cooling-warming events in the Quaternary glacial cycles^6^. Therefore, these cyclical events have played a dominant role in shaping the current distribution and genetic structure of many temperate species in the Northern Hemisphere^7^. East Asia has harboured the most diverse temperate flora in the world and was the most important glacial refuge for “Tertiary relics” throughout the Quaternary ice-age cycles. This region was never covered by massive ice-sheets during these glacial periods^8^; however, it experienced severe climatic oscillations throughout the Quaternary, with a dramatic effect on the evolution and distribution of both plants and animals^9^. Apart from historical factors such as Pleistocene range fragmentation and past demographic changes, more recent human-caused range fragmentation and isolation have also contributed to contemporary patterns of genetic variation within and between populations of species^10,11^. Evaluation of the relative influences of historical and contemporary elements on genetic diversity of forest trees has been a major topic in forest conservation for many years^5,12^.

Various approaches have been used to evaluate these influences, including using different types of analytical methods to test historical and contemporary gene flow or effective population sizes (*N*_e_) based on extant samples^13,14^. Recently, a very powerful and flexible approach, Approximate Bayesian computation (ABC), has been used to estimate demographic and historical parameters (e.g., effective population size, divergence time) and quantitatively compare alternative scenarios^15^. This approach can be used to resolve many important questions in ecology and evolutionary biology^15^. In addition, it should be emphasized that molecular markers with different rates of substitution can capture the signatures of historical and contemporary processes and could therefore help to determine historical demographic patterns^16–18^. Nuclear DNA (nDNA) is recombined, bi-parentally inherited and affected by both pollen and seeds, which can complicate attempts to reveal species phylogeographic, evolutionary history and gene flow patterns^19^. By contrast, chloroplast DNA (cpDNA) in angiosperms is usually maternally inherited, and thus, phylogeographical patterns revealed by cpDNA-based markers are solely due to seed dispersal^19,20^. In addition, the mutation rates in cpDNA are low and cpDNA markers are not affected by recombination^21,22^. Therefore, comparisons of genetic structure detected by nDNA and cpDNA marker analyses can also assess differences between pollen and seed flow dynamics. Furthermore, phylogeographic studies has been integrated ecological niche modelling (ENM), which provided relevant information about the geographic distributions of species or genetic lineages and their relationship with the spatial distribution of genetic diversity^23,24^.

Microsatellites (SSRs) are known to be highly polymorphic, codominant markers that have been widely used in studies of population genetics^25,26^. In this study, we first to analyse the population genetic structure and phylogeographic history of *L*. *auriculata* using nuclear (nSSR) and chloroplast (cpSSR) microsatellite markers to assess historical and contemporary evolutionary processes, investigate the causes of the endangerment of this species and provide basic guidelines for its conservation. Second, we also use approaches for genetic variation to detect changes in population size over different time scales in the present study. Third, we used ENM to infer the climatic potential niche of *L*. *auriculata* under current and Last Glacial Maximum (LGM) conditions. The aims of this study were as follows: (i) to evaluate the genetic structure of *L*. *auriculata*; (ii) to assess historical and contemporary evolutionary processes in shaping the genetic structure; (iii) to infer the species population demography using an ABC approach; (iv) to investigate how climatic and geographical variation over space and time to shape the patterns of genetic structure; and (v) to propose conservation and management of *L*. *auriculata* populations based on genetic structure and evolutionary history.

## Results

### Genetic diversity and network

The results of sequencing indicated that variations in allele size were due to the increase or decrease in the number of repeats. The seven cpSSR loci yielded a total of 20 alleles and 30 different haplotypes (H1 to H30), among 231 individuals of *L*. *auriculata*. The number of haplotypes per population yielded 3 to 17 haplotypes in the eight populations, and the mean number of haplotypes for the eight populations was 6.5. The most polymorphic populations were seen in AY in Anhui province, with 17 haplotypes. The HY population had only 3 haplotypes. The most common haplotypes were H1 (24.7%) and H2 (26.5%), found in all populations, followed by H6 (11.4%) and H7 (11.9%). The haplotype H2 (26.5%) showed the highest frequency in all populations. A high level of genetic diversity at the population level was detected. The number of different alleles (*N*_a_) ranged from 1.571 to 2.429, and the number of effective alleles (*N*_E_) ranged from 1.112 to 1.590 (Table 1). The average Shannon’s information index (*I*), diversity (*h*) and unbiased diversity (*uh*) values were 0.268 (0.160–0.511), 0.159 (0.088–0.315) and 0.167 (0.090–0.323), respectively (Table 1). Overall, the cpSSR data revealed high within-population diversity (*h*_S_ = 0.667, *v*_S_ = 0.698) and total diversity (*h*_T_ = 0.855, *v*_T_ = 0.851) in *L*. *auriculata* populations.

**Table 1:** Population genetic diversity revealed by nuclear and chloroplast microsatellite markers for *Litsea auriculata* eight populations.

Population	nSSR								cpSSR						
	N	*A*	*A*_R_	*P*_A_	*H*_o_	*H*_E_	*F*_IS_	*N*_e_	*N*_a_	*N*_E_	*I*	*h*	*uh*	*P* (%)	Haplotype
**Zhejiang Province**															
ZT	34	6.13	3.79	0.13	0.422	0.568	**0**.**271**	27.7	1.571	1.112	0.160	0.088	0.091	57.14	H1(24), H2(4), H3(1), H4(1), H5(1), H6(1)
ZQ	22	5.50	3.76	0.13	0.506	0.560	**0**.**119**	9.8	1.857	1.153	0.221	0.117	0.123	71.43	H1(1), H2(2), H7(13), H8(2), H9(1), **H10**(**1**), **H11**(**1**)
ZD	39	6.38	4.16	0.13	0.480	0.642	**0**.**265**	23.9	2.143	1.250	0.281	0.158	0.162	71.43	H1(1), H2(16), H7(11), H8(1), H9(1), H12(1), **H13**(**2**), **H14**(**2**), H15(1), **H16**(**1**)
**Anhui Province**															
AS	26	4.88	3.40	0.25	0.443	0.519	**0**.**166**	5.9	1.714	1.296	0.286	0.182	0.189	57.14	H1(3), H2(1), H4(1), H6(8), H7(1), H17(8), **H18**(**3**), H19(1)
AY	40	5.50	3.53	0.38	0.484	0.506	0.056	20.4	2.429	1.590	0.511	0.315	0.323	71.43	H1(2),H2(1), H3(1), H4(2), H5(1), H6(15), H12(2), H17(1), H19(1), **H20**(**1**), **H21**(**2**), **H22**(**1**), **H23**(**1**), **H24**(**1**), **H25**(**1**), **H26**(**3**), H27(1)
**Henan Province**															
HX	38	5.63	3.03	0.38	0.329	0.436	**0**.**258**	13.0	1.857	1.172	0.203	0.115	0.118	85.71	H1(9), H2(24), H6(1), H7(1), H27(2), **H28**(**1**)
HJ	25	4.38	3.04	0.13	0.378	0.483	**0**.**237**	4.2	1.714	1.191	0.208	0.122	0.128	71.43	H1(11), H2(7), H8(1), **H29**(**1**), **H30**(**1**)
HY	7	3.38	3.19	0.00	0.396	0.404	0.098	3.2	1.571	1.276	0.273	0.175	0.204	57.14	H1(3), H2(3), H15(1)
Mean	23.3	5.22	3.49	0.19	0.430	0.515	0.184	13.5	1.857	1.255	0.268	0.159	0.167	67.86	

*A*: mean number of alleles per locus. *A*_R_: allelic richness. *P*_A_: number of private alleles for all loci. *H*_O_ and *H*_E_ are mean observed and expected heterozygotes for all loci, respectively. *F*_IS_: mean inbreeding coefficients for all loci, with *F*_IS_ in bold indicating significant deviation from zero with a sequential Bonferroni correction. *N*_e_: effective population size. *N*_a_: number of different alleles. *N*_E_: number of effective alleles. *I*: Shannon’s information index. *h*: diversity. *uh*: unbiased diversity. *P*: percentage of Polymorphic Loci. Haplotypes in bold are rare.

**Figure 1:**
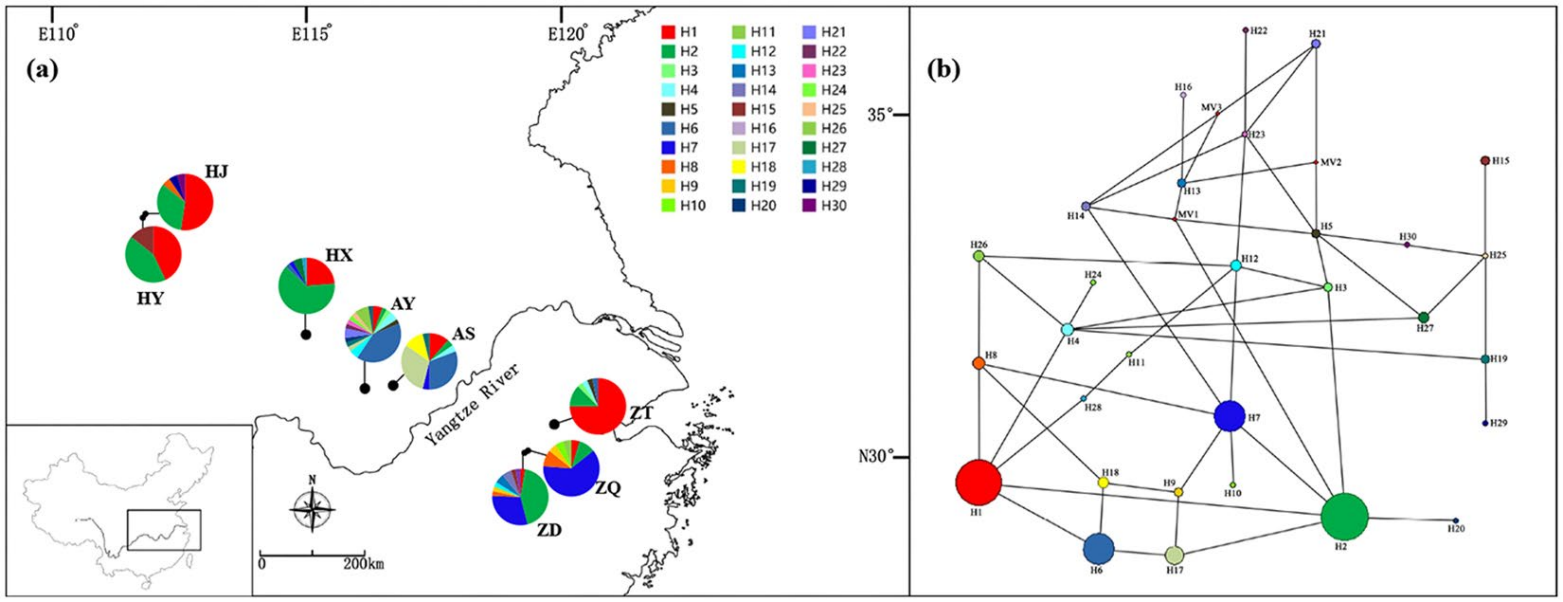
Map of China showing the sampling locations and geographic distribution of the chloroplast haplotypes found in *Litsea auriculata* populations [The original map was created by using Google Earth Pro 7.1.2.2041, which was free downloaded from https://google-earth-pro.en.softonic.com/ and the edge of China was drew by using AutoCAD 2010 (https://www.autodesk.com/education/free-software/autocad)]. (**a**) Pie charts on the map represent the haplotype composition of samples from the corresponding populations. The colour in each chart represents the haplotype, as indicated in the cluster tree; (**b**) Cluster analysis of 30 haplotypes detected in 231 samples based on the median-joining haplotype network developed using NETWORK 5.0. Haplotype circle sizes are proportional to the frequency of each Haplotype.

Median-joining haplotype network reflecting topology and haplotype frequency was constructed for cpSSRs (Fig. 1). The distribution of haplotypes of *L*. *auriculata* showed no obvious geographic structure (Fig. 1). These results were further confirmed as the *R*_ST_ (0.180) was significantly less than *G*_ST_ (0.221) (*P* < 0.05), indicating a lack of significant phylogeographic structure in *L*. *auriculata* populations.

**Figure 2:**
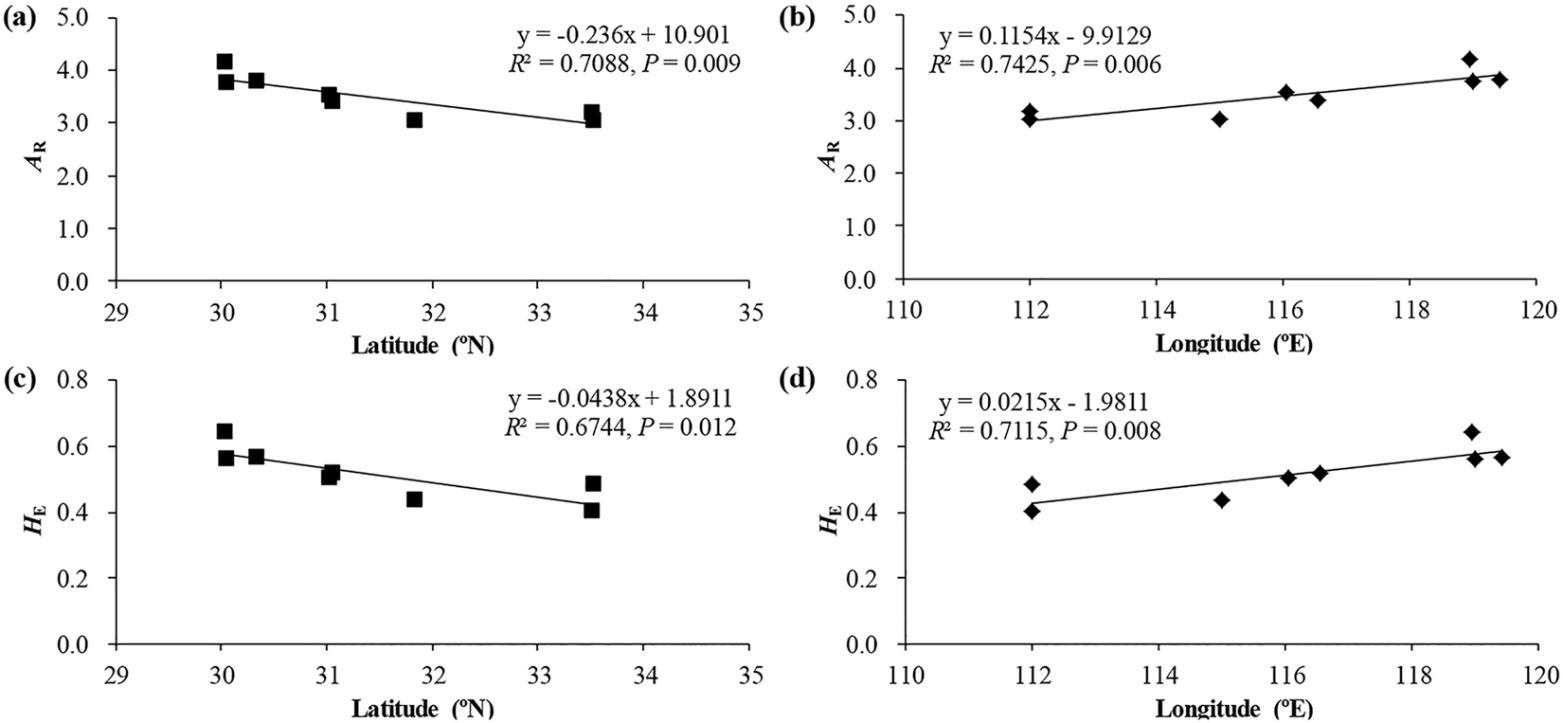
Population genetic parameters [allelic richness (*A*_r_) and expected heterozygosity (*H*_E_)] in relation to geographic coordinates (latitude and longitude).

Across all nuclear microsatellite loci, a total of 76 alleles were detected, and high levels of gene diversity (*H*_T_ = 0.657, *H*_S_ = 0.530) were observed. Genetic diversity at the population level, the mean number of alleles per locus (*A*) and the allelic richness (*A*_R_) for each population ranged from 3.38 to 6.38 and from 3.03 to 4.16, respectively (Table 1). The observed (*H*_O_) and expected (*H*_E_) heterozygosity for each population ranged from 0.329 to 0.506 and from 0.404 to 0.642, respectively (Table 1). The mean number of alleles and expected heterozygosity for each population were highest in ZD (*A* = 6.38, *H*_E_ = 0.642) and lowest in HY (*A* = 3.38, *H*_E_ = 0.404). In total, 34 private alleles (*P*_A_) were detected within eight populations, and *P*_A_ ranged from 0.00 to 0.38 in eight populations. The highest *P*_A_ values were found in AY and HX populations. Inbreeding coefficients (*F*_IS_) ranged from 0.098 to 0.271 (Table 1), six of which significantly deviated from zero indicating that most of the populations experienced inbreeding. Effective population size (*N*_e_) values for all *L*. *auriculata* populations were less than 50 (Table 1). The values of allelic richness (*A*_r_) and expected heterozygosity (*H*_E_) were significantly negative correlated to the latitude (*R* ^2^ = 0.709, *P* = 0.009 and *R* ^2^ = 0.674, *P* = 0.012; Fig. 2), while there was significantly positive correlation to the longitude (*R* ^2^ = 0.743, *P* = 0.006 and *R* ^2^ = 0.712, *P* = 0.008; Fig. 2).

### Genetic structure

The AMOVA tests indicated significant differentiation among all populations. For the cpSSR data, 28.54% of the total variation was attributed to differences among populations, and 71.46% within populations (Table 2). For nSSR, 16.50% of the total variation was attributed to differences among populations, and 83.5% within populations (Table 2). Relatively high genetic differentiation among the eight populations (*F*_ST(c)_ = 0.285, wright *F*_ST(n)_ = 0.165) was also observed based on cpSSR and nSSR data, respectively. Pairwise *G*_ST_ values based on cpSSR data among populations ranged from 0.004 to 0.220. The highest *G*_ST_ value was found between populations ZQ and AS (*G*_ST_ = 0.220), and the lowest between populations HJ and HY (*G*_ST_ = 0.004) (Table 3). Meanwhile, pairwise *F*_ST_ values based on nSSR data among populations ranged from 0.061 to 0.340. The highest *F*_ST_ value was observed between populations HX and HY (*F*_ST_ = 0.340), and the lowest between populations HJ and HY (*F*_ST_ = 0.061, Table 3). All *G*_ST_ and *F*_ST_ estimates were significant (*P* < 0.05, Table 3) except for that between the two populations HJ and HY, which confirmed that genetic differentiation among the populations was quite high.

**Table 2:** Analysis of molecular variance (AMOVA) in nuclear and chloroplast microsatellite loci across *Litsea auriculata* populations.

	Source of variation	d.f.	Sum of square	Variance components	Percentage of variation (%)	Fixation indices	*P* value
cpSSR	Among populations	7	50.140	0.236	28.54	*G*_ST_ = 0.285	0.000
	Within populations	223	129.742	0.590	71.46		
	Total	231	179.882	0.826			
nSSR	Among populations	7	13646.722	31.874	16.50	*F*_ST_ = 0.165	0.000
	Within populations	223	72310.851	161.251	83.50	*F*_IS_ = 0.027	0.000
	Total	231	85957.573	193.125		*F*_IT_ = 0.187	0.000

**Table 3:** Pairwise genetic differentiation between *Litsea auriculata* populations.

Populations	ZT	ZQ	ZD	AS	AY	HX	HJ	HY
ZT	—	0.209	0.126	0.089	0.057	0.038	0.010	0.018
ZQ	0.113	—	0.033	0.220	0.198	0.127	0.156	0.129
ZD	0.100	0.076	—	0.160	0.140	0.041	0.074	0.058
AS	0.183	0.234	0.150	—	0.052	0.104	0.097	0.103
AY	0.180	0.260	0.177	0.149	—	0.087	0.072	0.066
HX	0.247	0.324	0.247	0.149	0.244	—	0.013	0.015
HJ	0.210	0.190	0.162	0.265	0.281	0.340	—	0.004^NS^
HY	0.190	0.179	0.174	0.310	0.327	0.361	0.061^NS^	—

Above diagonal, Gregorius (1974) genetic distance using a binary matrix of cpSSR haplotype data; below diagonal, *F*_ST_ measured from nuclear SSR, NS indicate non-significant population differentiation where *P* > 0.05.

**Figure 3:**
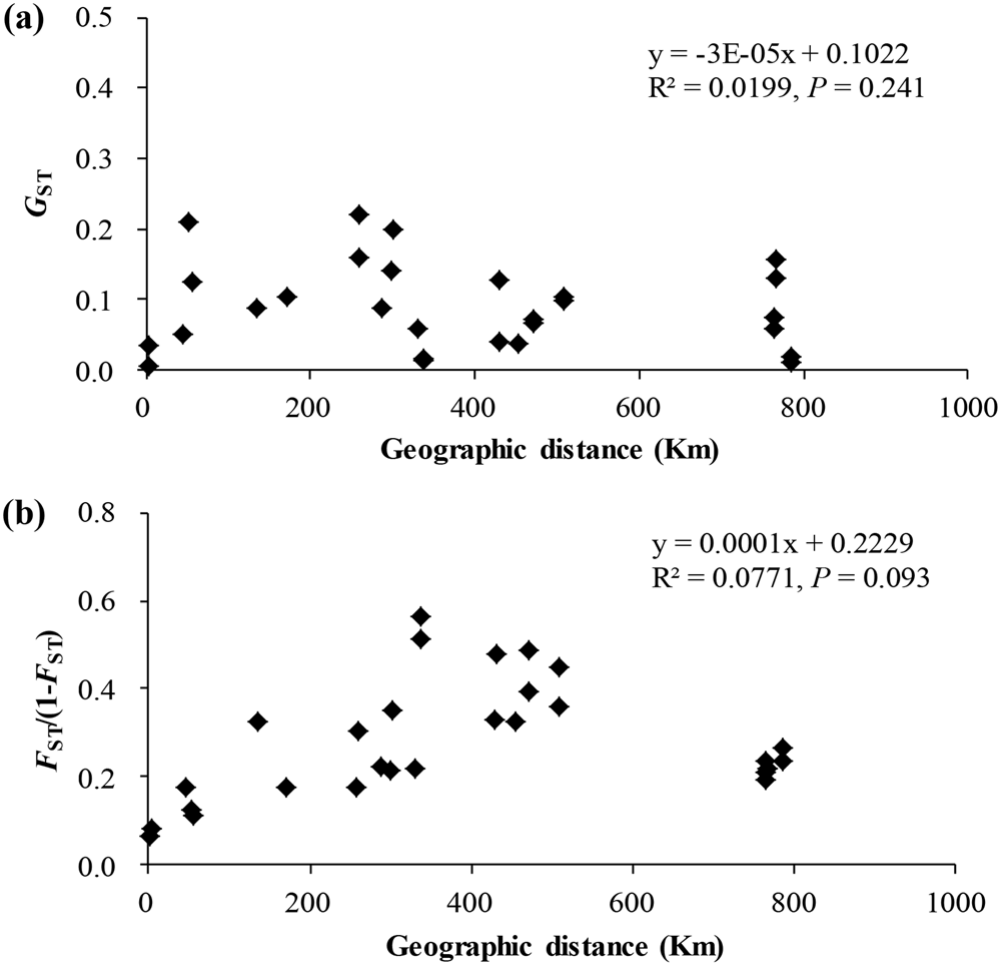
(**a**) Correlations between Nei’s unbiased genetic distance (*G*_ST_) estimated by cpSSR loci and the geographical distance of *Litsea auriculata* populations. (**b**) Correlation between the genetic *F*_ST_/(1− *F*_ST_) estimated by nuclear SSR loci and geographic distances among *Litsea auriculata* populations.

The mantel test indicated that no significant correlations between geographic and genetic distance were observed from the cpSSR (y = −3E-05x + 0.1022, R^2^ = 0.0199, *P* = 0.241) and nSSR (y = 0.0001x + 0.2229, R^2^ = 0.0771, *P* = 0.093) analysis (Fig. 3).

**Figure 4:**
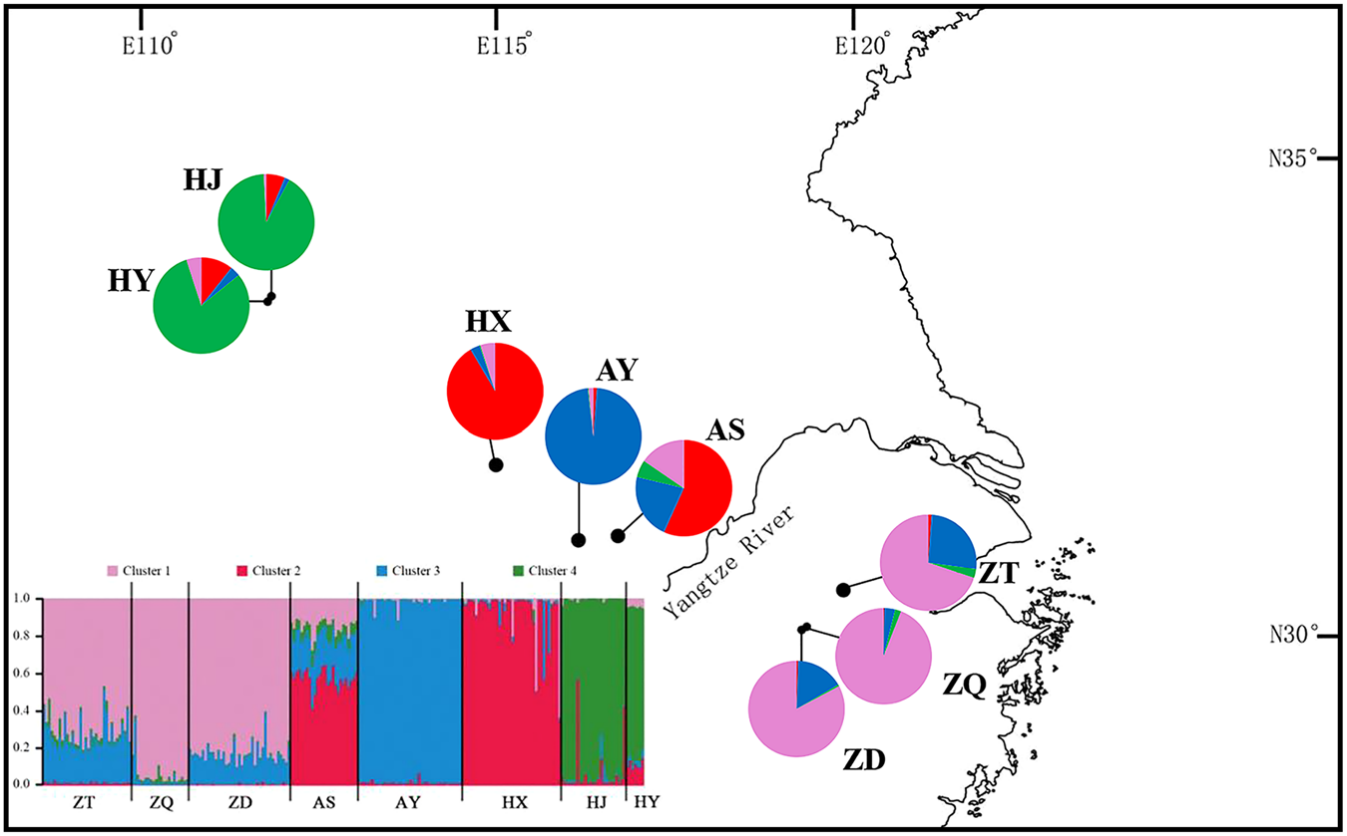
Geographic distribution of the genetic groups detected from STRUCTURE analysis of *Litsea auriculata* (Δ*K* = 4) [The original map was created by using Google Earth Pro 7.1.2.2041, which was free downloaded from https://google-earth-pro.en.softonic.com/ and the edge of China was drew by using AutoCAD 2010 (https://www.autodesk.com/education/free-software/autocad)] .

In the STRUCTURE analysis of *L*. *auriculata* using eight nSSR loci, the ad hoc statistic ∆*K* recovered the highest likelihood at *K* = 4 (Supplementary Fig. S1). All *L*. *auriculata* individuals were assigned to four clusters (Fig. 4). Cluster I (pink) contained ZD, ZT and ZQ populations from Tianmu Mountain. Cluster II (red) included AS and HX populations and AS showed evidence of extensive admixture. Cluster III (blue) only contained the AY population and Cluster IV (green) contained the remaining two populations (HJ and HY) from Qinglin Mountain.

**Figure 5:**
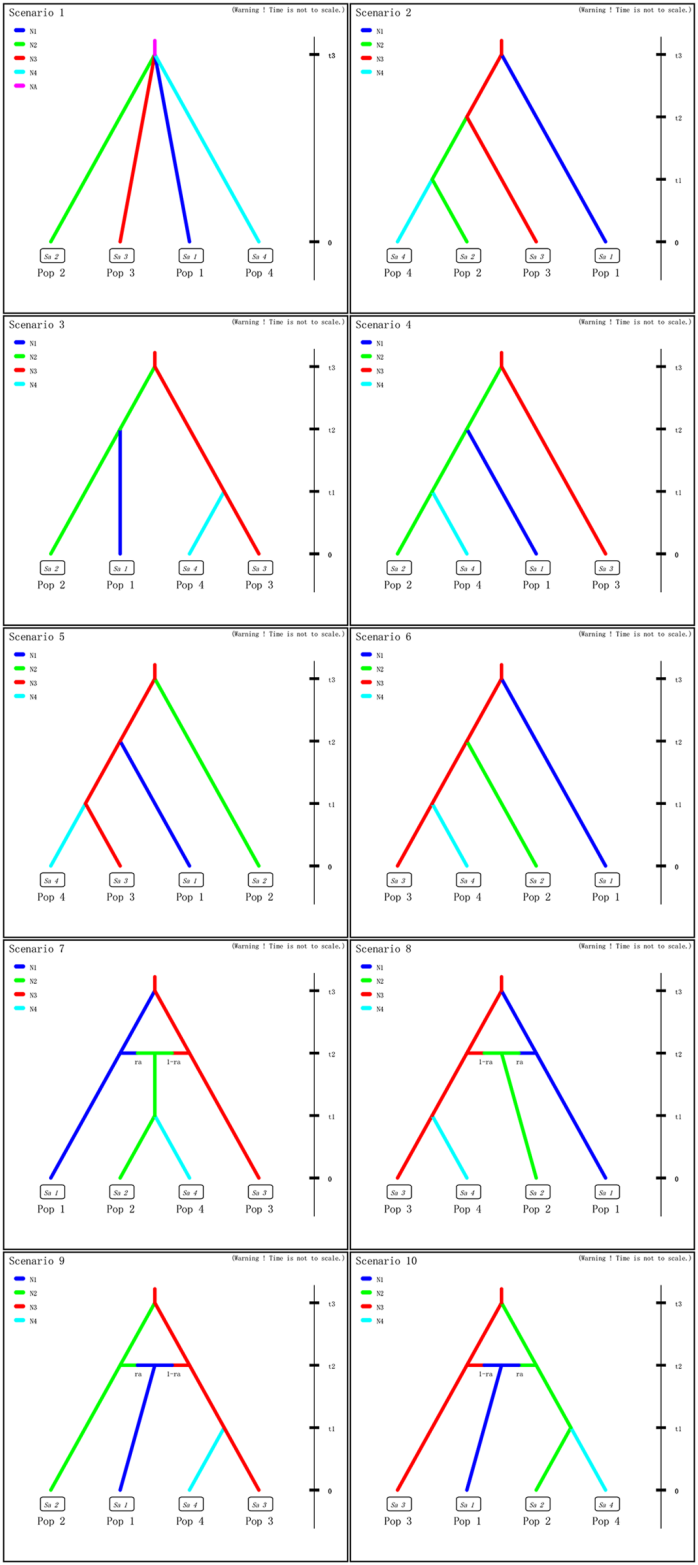
Ten demographic scenarios of *Litsea auriculata* assessed using DIYABC. Time in generations is *t* (*t*3 ≥ *t*2 ≥ *t*1).

### ABC-based inferences of population divergence

Among the ten predesigned scenarios in DIYABC analysis (Fig. 5), scenario 1 had the highest posterior probability (0.9767, 95% CI: 0.9745, 0.9788); it was much higher than other scenarios and did not overlap with those obtained for the other scenarios (Table 4, Supplementary Fig. S2). Based on model performance, we discriminated scenario 1 from the other scenarios. This is corroborated by model checking: PCA showed that the observed data point was centred around the cluster of points for the simulated data based on posterior distributions (Supplementary Fig. S3), and no summary statistics showed a significant difference between the observed and simulated data based on posterior distributions (Supplementary Table S1). This suggested that scenario 1 was still fitted to the observed data. Analyses to estimate confidence in scenario choice, based on 500 PODs, indicated that type I and Type II errors for the best-supported scenario 1 were 0.038 and 0.036, respectively; these are low errors. Under scenario 1, posterior mean parameter estimates indicated that the simultaneous divergence occurred 3,010 (95% CI: 519–10 900) generations ago, corresponding to 60,200 years (95% CI: 10,380–218,000 years with a generation time of 20 years). For scenario 1, the median values of the effective population size were 29,600 (95% CI: 6,610–86,900) for N1 (ZT, ZQ and ZD population), 19,600 (95% CI: 3,930–78,100) for N2 (AS and HX population), 14,000 (95% CI: 2,720–70,200) for N3 (AY population), 9,210 (95% CI: 1,720–61,200) for N4 (HJ and HY population), and 3,020 (95% CI: 127–37,900) for NA before the divergence at t3 (Supplementary Fig. S4, Supplementary Table S2). The posterior parameter estimation indicated that the effective population size of ancestral populations were hundreds of times higher than those of current populations (*N*_e_ = 13.5), which suggest that species have undergone major contraction of their ranges. Moreover, the N1 group has the largest effective population size, and the N4 group has the smallest effective population size (N1 > N2 > N3 > N4). The estimated mean mutation rate of nSSRs was 1.74 × 10^−4^ (95% CI: 1.05 × 10^−4^–5.65 × 10^−4^).

**Table 4:** Posterior probabilities of each scenario and 95% confidence intervals tested by approximate Bayesian computation analyses (ABC) on nSSRs data.

Scenarios	Posterior probability	95% Credibility Interval
1	0.9767	[0.9745,0.9788]
2	0.0039	[0.0032,0.0045]
3	0.0009	[0.0008,0.0011]
4	0.0051	[0.0043,0.0060]
5	0.0014	[0.0012,0.0017]
6	0.0012	[0.0010,0.0013]
7	0.0050	[0.0042,0.0058]
8	0.0011	[0.0010,0.0013]
9	0.0006	[0.0005,0.0007]
10	0.0040	[0.0034,0.0047]

### Contemporary and historical gene flow

The results of the migration rates estimated by BAYESASS suggested a significantly lower level of contemporary gene flow (*m*_c_) throughout all populations. The average migration rates in all pairwise comparisons ranged from 0.007 to 0.176, with standard deviations less than 0.05 (Table 5). Each value represents the proportion of individuals derived from a corresponding source population for each generation. However, historical migration rates (*m*_h_) estimated by Migrate-N were significantly high and ranged from 0.037 (ZD to HJ) to 0.367 (from HJ to HY) (Table 6). Estimates of contemporary gene flow demonstrated a lack of significant gene flow among populations, which suggesting that gene flows were affected by the recent habitat fragmentation of *L*. *auriculata*.

**Table 5:** Recent migration rate estimated from BAYESASS across eight populations of *Litsea auriculata*

	ZT	ZQ	ZD	AS	AY	HX	HJ	HY
ZT	—	0.010	0.010	0.016	0.011	0.009	0.011	0.011
ZQ	0.034	—	0.015	0.012	0.019	0.011	0.011	0.011
ZD	0.013	0.014	—	0.015	0.013	0.013	0.008	0.008
AS	0.011	0.018	0.011	—	0.012	0.012	0.010	0.010
AY	0.008	0.007	0.008	0.010	—	0.012	0.007	0.007
HX	0.007	0.021	0.007	0.008	0.011	—	0.007	0.007
HJ	0.010	0.012	0.011	0.011	0.010	0.019	—	0.010
HY	0.024	0.022	0.023	0.022	0.022	0.022	0.176	—

The first column represents the destination population, while the first row represents the origin population. Values in bold are the proportion of individuals derived from the source population each generation.

**Table 6:** Historical migration rate estimated from Migrate-N across eight populations of *Litsea auriculata*

	ZT	ZQ	ZD	AS	AY	HX	HJ	HY
ZT	—	0.126	0.041	0.099	0.102	0.082	0.199	0.072
ZQ	0.106	—	0.246	0.085	0.103	0.141	0.131	0.070
ZD	0.106	0.066	—	0.113	0.158	0.037	0.144	0.091
AS	0.127	0.072	0.220	—	0.176	0.181	0.099	0.094
AY	0.098	0.053	0.160	0.108	—	0.090	0.131	0.096
HX	0.097	0.102	0.132	0.133	0.118	—	0.078	0.081
HJ	0.113	0.099	0.042	0.044	0.179	0.064	—	0.146
HY	0.367	0.157	0.090	0.110	0.084	0.110	0.251	—

The first column represent the population of destination, while the first row represent the population of origin. Values in bold are the proportion of individuals derived from the source population each generation.

### Population bottleneck test

The inbreeding coefficients ranged from 0.056 to 0. 265, and all eight populations showed significant deviation from mutation-drift equilibrium under IAM assumptions and a mode-shift test (Table 7). Furthermore, six (ZT, ZD, AY, HX, HJ and HY) and three populations (AY, HX and HY) also showed significant deviation from mutation-drift equilibrium under SMM and TPM assumptions, respectively (Table 7). These results indicate that all eight populations appear to have undergone a recent bottleneck, resulting in a significant excess in heterozygosity compared with the heterozygosity expected.

**Table 7:** Sample locations and two-tailed *P* values for Wilcoxon signed-rank test for heterozygozity excess or deficiency under three mutation models.

Population	Location	Latitude (N)	Longitude (E)	Altitude (m)	IAM	SMM	TPM	Mode-shift test
**Zhejiang Province**								
ZT	West Tianmu Mountain, Linan City	N30°20.393′	E119°25.827′	1067	0.004*	0.039*	0.547	Shifted
ZQ	Baishuiwu, Qingliangfen, Linan City	N30°2.899′	E119°0.09′	995	0.055*	0.195	0.195	Shifted
ZD	Xikeng, Daming Mountain, Linan City	N30°2.213′	E118°58.384′	980	0.008*	0.027*	0.055	Shifted
**Anhui Province**								
AS	Shucheng, Lu’an City	N31°3.388′	E116°32.93′	576	0.027*	0.195	0.313	Shifted
AY	baojia Town, Yuexi, Anqing City	N31°1.89′	E116°4.183′	1056	0.004*	0.004*	0.004*	Shifted
**Henan Province**								
HX	Jigong Mountain, Xinyang City	N31°50.502′	E114°59.991′	476	0.004*	0.004*	0.008*	Shifted
HJ	Jiuya village, Qiaoduan, Nanyang City	N33°32.140′	E112°0.860′	741	0.004*	0.008*	0.074	Shifted
HY	Yuzang village, Qiaoduan, Nanyang City	N33°31.125′	E112°0.042′	840	0.008*	0.008*	0.008*	Shifted

*Significant excesses (in two-tailed Wilcoxon test) in gene diversity compared with expected gene diversity at mutation-drift equilibrium. Under the mode-shift test, a distribution with a shifted mode is expected in a population that has undergone a bottleneck.

### Ecological niche modelling

**Figure 6:**
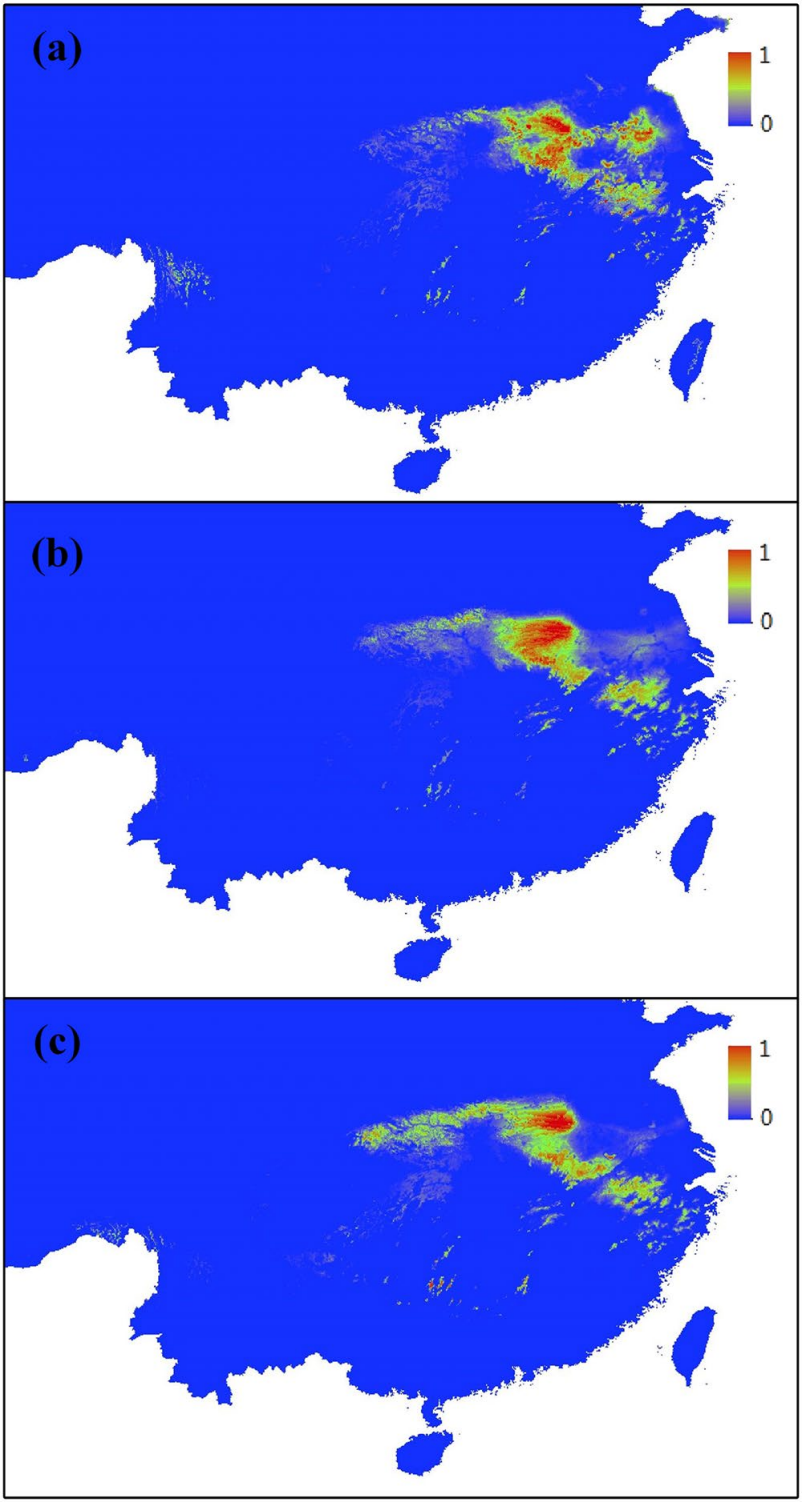
Predicted distribution of *Litsea auriculata* based on Ecological niche distribution model. (**a**) Predicted distribution based on current data; (**b**) distribution during the Last Glacial Maximum (LGM) based on community climate system model (CCSM); (**c**) distribution during LGM model based on model for interdisciplinary research on climate (MIROC). Ecological niche models were established with current bioclimatic variables on the basis of extant occurrence points of the species using Maxent version 3.4.1. Predicted distribution probabilities are shown in each 2.5 arc-min pixel. The map of China is free downloaded from Global Administrative Areas (http://www.gadm.org/country). The map is made by ArcGIS 10.3 software (http://www.arcgis.com/features/index.html), and then cut by Visio Pro for Office 365 (Trial) (https://products.office.com/zh-cn/visio/visio-professional-free-trial-flowchart-software).

ENM were reconstructed for distribution of the present and the LGM (Fig. 6a–c). Models performance showed that the average values of all models are 0.998, indicating good performance of the models. The potential distribution for the present range of *L*. *auriculata*, predicted by Maxent, is very similar to the actually known geographic distribution of the species (Fig. 6a) in the central-east China. Palaeodistribution modelling suggested a relatively larger area during the LGM than that predicted under current conditions (319 grid numbers, *P* ≥ 0.75), based on both CCSM (425 grid numbers, *P* ≥ 0.75) and MIROC (608 grid numbers, *P* ≥ 0.75, Fig. 6b,c). The CCSM indicated that optimum suitable habitat was in the central-east China, which was decreased after LGM about 24.9%. Based on MIROC, the range decreased 47.5%. Both models suggested that central-east China experienced habitat contraction and declines after LGM, although at different levels, which suggest that species experienced considerable population decreases, and suffered many population extinctions. The suitable distribution of *L*. *auriculata* flowed from the central-east area to the southeast of China (Fig. 6), which may contribute to the climate change. The potential distribution of current was more scattered, while LGM was more concentrated based on two models (Fig. 6). In addition, the suitable distribution area of *L*. *auriculata* is separated by the Yangtze River from LGM to current (Fig. 6).

## Discussion

### Population demography relative to climatic changes

Based on the coalescent theory, the most frequent and widespread haplotypes are likely to be the ancestral haplotypes^27^. Median-joining haplotype network analyses revealed that haplotype H1 and H2 may be ancestral haplotypes, which are distributed in all populations. The widespread occurrence of ancestral haplotypes (H1 and H2) appeared among the widely disjunct populations (Table 1, Fig. 1), which may result from historical migration events and/or shared ancestral polymorphism due to incomplete lineage sorting rather than long-distance gene flow^28^. Similar conclusions have also been drawn for other species, such as *N*. *sericea* ^28^.

Mainland China, as part of the Sino-Japanese Floristic Region, harbours the most diverse of the world’s temperate flora and was the most important glacial refuge for its Tertiary representatives (“relics”) throughout Quaternary ice-age cycles^9,29,30^. Although this region has never been directly impacted by extensive ice sheets^29,31^, it experienced severe climatic oscillations throughout the Quaternary, resulting in repeated drastic environmental changes that profoundly shaped the current distributions and genetic structures of many plant and animal species^32,33^. The results of the phylogeographic analysis are consistent with the basic Quaternary expansion-contraction (EC) model, which also proved to be consistent with fossil pollen evidence in indicating extensive latitudinal range shifts, typically in the form of southward retreats during glacial events, followed by rapid expansions northward during inter-/postglacial events^9,34,35^. The tropical southern populations from potential refugia perhaps maintain higher levels of genetic diversity, consistent with the EC model^9^. The climatic fluctuations throughout inter-/postglacial events would have altered the distribution of habitats for *L*. *auriculata*, leading to variation in population continuity and isolation. The values of *H*_E_ and *A*_r_ were higher in the southern area than the northern area, and higher in the eastern area than western area. These values significantly decreased as latitude increased, but increased as longitude increased in the present study (Fig. 2). This result is consistent with the reconstruction of species distribution at LGM.

The STRUCTURE analysis revealed that eight *L*. *auriculata* populations were assigned to four groups (Fig. 4). These four groups located at three mountains distant from each other or between the Yangtze River and diverged at the same time (60,200 years), corresponding to the beginning of the last ice age; this was strongly supported by the ABC analyses. This scenario is also consistent with the reconstruction of species distribution at LGM, which is supported by both CCSM and MIROC (Fig. 6b,c). The models predicted the occurrence of discontinuous suitable areas between south (Tianmu Mountain) and north regions (Dabie Mountain, Qinling Mountain and Funiu Mountian) along both banks of Yangtze River (Fig. 6). Moreover, it is notable that paleoenvieronmental reconstructions also showed that the suitable distribution of *L*. *auriculata* was scattered especially based on MIROC model (Fig. 6c). Although mainland China was not covered by ice sheets during the ice age, the four groups of *L*. *auriculata* occupy geographic mountains and Yangtze River separated by habitat type, driving isolation and genetic divergence between groups of populations enhanced by environmental differences.

In the genetic pattern observed, we also found the signature for past demographic changes in *L*. *auriculata* populations. The demographic history inferred from the ABC analyses is that the four groups expansion starting about 60,200 years BP, that is, during the last glacial phase including the LGM. Although the lack of a calibrated molecular clock demands caution, paleoenvironmental reconstructions of ENM at the LGM support the hypothesis that populations of this species expanded during this period.

ENM of habitat changes following the LGM show clearly that the current distribution of *L*. *auriculata* experienced severe habitat shifting and fragmentation, which is supported by both CCSM and MIROC (Fig. 6). The suitable distribution of *L*. *auriculata* experienced a northwest-to-southeast migration in China and scattered. Compare the ENM of LGM and current, AY population (Dabie Mountian) exists in optimum suitable habitats wherever in LGM or current, which contains the highest haplotype diversity, with the most (unique) haplotypes and private alleles (Table 1). Moreover, Dabie Mountains is the north passageway of plant spread, where floristic elements can spread from southwest China and central China to east China or from east China to west China and central China^36^.

Our study supports hypothesis that range and distribution of suitable habitat are the key factors determining demographic histories. The suitable habitats of a species are influenced by both climatic and geographic features (such as Yangtze River, Mountains). Climate fluctuations have enormous impacts on changes in a species’ suitable habitat, however these impacts are also influenced by geographic features. This result supports the idea that climatic and geographic features can impact the evolutionary history of a species.

### Impact of habitat fragmentation on *L*. *auriculata* populations

Our ABC and ENM analysis through time suggested that populations likely experienced expansion and retraction into and out of local refugia, and Quaternary climatic fluctuation may have played role in genetic structure as well^9,37^. As a result, the distribution area of *L*. *auriculata* is drastic decreased and scattered in several mountains along the Yangtze River. Therefore, *L*. *auriculata* populations were expected to experience erosion of genetic variation, significant population bottlenecks, reduced remnant population size and increased population genetic divergence due to increased random genetic drift, high levels of inbreeding and reduced gene flow^38,39^. In the present study, *L*. *auriculata* did exhibit genetic signs of a high level of population divergence (*F*_ST(c)_ = 0.285, wright *F*_ST(n)_ = 0.165), a significant population bottleneck (Table 7), a relatively small effective population size (*N*_e_ = 13.5), significant increases in inbreeding coefficient (*F*_IS_ = 0.184) and reduced gene flow—i.e., the contemporary gene flow (*m*_c_: 0.007–0.176) is lower than historical gene flow (*m*_h_: 0.037–0.367) in *L*. *auriculata* populations. Nevertheless, high levels of genetic diversity were observed in *L*. *auriculata* at both the species (*h*_T_ = 0.855, 30 haplotypes based on cpSSR loci; *H*_T_ = 0.657 based on nSSR loci) and population level (*H*_E_ = 0.515, *h* = 0.159).

The significant population bottlenecks may be explained by a recent decline in population size due to habitat destruction. Eight populations appear to have undergone recent bottlenecks (Table 7), resulting in a significant excess of heterozygosity. The number of alleles decreases more rapidly than heterozygosity during a population decline, this will result in an excess heterozygosity for this particular population^40^. This is consistent with increasing human activity during the past century, including agriculture, urbanization and deforestation^2^, which resulted in a dramatic reduction in the number of sites and population sizes of *L*. *auriculata*. In addition, in the present study estimates for *N*_e_ values were all less than 30, with a mean of 13.5 (Table 1). Although different populations may lose different alleles, a large number of alleles may still be maintained across the species as a whole^41^. However, these *L*. *auriculata* populations remain relatively rich in genetic diversity (at species level: *h*_T_ = 0.855, 30 haplotypes based on cpSSR loci; *H*_T_ = 0.657 based on nSSR loci; at population level: *H*_E_ = 0.515, *h* = 0.159) even when experiencing drastic habitat fragmentation. Genetic variation may decrease with reduced remnant population size, but not all fragmentation events lead to genetic losses and different types of genetic variation^38^. Thus, genetic drift does not appear to lead to decreased genetic diversity, but still increased population differentiation^42^. Furthermore, *L*. *auriculata* is a perennial species, and this habit may delay time between generations and buffer the effect of habitat fragmentation on genetic erosion^43^. Similar results were found in other endangered species in the same family, such as *L*. *szemaois* (AFLP: *H*_T_ = 0.1947; ISSR: *H*_T_ = 0.2498) in SW China^42^, *Machilus thunbergii* (*H*_T_ = 0.165, 9 haplotypes) in Southeast Asia^44^, *M*. *kusanoi* (*H*_T_ = 0.159, 9 haplotypes) in southeast China^44^, and *Neolitsea sericea* (*H*_T_ = 0.761, 9 haplotypes) in Southeast Asia^28^.

Contemporary gene flow (*m*_c_ = 0.007–0.176) among populations was lower than historical gene flow (*m*_h_ = 0.037–0.367, Table 5). This finding demonstrates that the recent habitat fragmentation in these *L*. *auriculata* populations is accompanied by decreased gene flow. This reduced gene migration between *L*. *auriculata* populations (Tables 5 and 6) might be attributed to poor dispersal ability, combined with isolation; and this is consistent with expectations for insect-pollinated and fleshy-fruited (bird-mediated seed dispersal) species, for which pollen and seed dispersal is relatively limited^45^. This conclusion was confirmed by several studies in Lauraceae, such as *L*. *szemao* ^42^ and *Neolitsea sericea* ^46,47^. Reduced contemporary gene flow can further decrease genetic diversity and contribute to high levels of among-population genetic differentiation.

Another expectation for fragmentation population is increased inbreeding (*F*_IS_ = 0.184), which is influenced by the mating system and the limited gene flow^48^. We found that inbreeding coefficients ranged from 0.056 to 0.265, and all populations significantly deviated from mutation-drift equilibrium under IAM assumptions and a mode-shift test (Table 7), indicating that all *L*. *auriculata* populations experienced inbreeding. Since *L*. *auriculata* is a dioecy species with insect-pollinated outcrossing trees, inbreeding could be due to mating between relatives, such as half-sibs, rather than selfing^49^.

Fragmentation usually decreases the habitat sizes and increases spatial distances between populations, leading to high levels of genetic differentiation (*F*_ST(c)_ = 0.285, wright *F*_ST(n)_ = 0.165). Similar results have been reported in other endangered or endemic species of the same family, such as *L*. *szemaois* (*Φ*_st_ = 0.2420)^42^ and *N*. *sericea* (*F*_ST(c)_ = 0.355; *F*_ST(n)_ = 0.141)^28^. In addition, hierarchical AMOVA (28.54% for cpSSR and 16.50% for nSSR, Table 2) also showed the similar level of genetic differentiation among populations. Furthermore, all pairwise *G*_ST_ and *F*_ST_ values among populations were also significant (*P* < 0.05, Table 3) except between HY and HJ, which further confirmed that genetic differentiation among populations was quite high. Meanwhile, a lack of significant correlations between geographic and genetic distance based on cpSSR and nSSR analysis were detected (Fig. 3), which may be attributed to a higher impact of genetic drift. At very small spatial scales gene flow may not be sufficiently unidirectional to cause a significant correlation^50^.

### Conservation implications

*L*. *auriculata* is listed in the “Lower Risk/near-threatened” category of the IUCN red list of threatened species, which means that this species is close to being qualified as vulnerable. Information on the genetic diversity, genetic structure, and demographic history of endangered species intergrate with ecological niche modeling can establish effective and efficient strategies for their conservation. One of our aims for the conservation of threatened plants is to preserve their genetic diversity^51^. Although *L*. *auriculata* has a relatively higher level of genetic diversity, its distribution, population size and effective population size are fairly small. Therefore, this species faces an extinction risk in the short term. An effective population size (*N*_e_) greater than 50 is proposed to prevent inbreeding depression in the short-term (5 generations) and a *N*_e_ greater than 500 was proposed to maintain the evolutionary potential in perpetuity^52^. However, all of the estimated *N*_e_ in populations of *L*. *auriculata* were less than 30 (Table 1). Therefore, we suggest that protection zones in the distribution areas of *L*. *auriculata* should be established to protect the habitat for this species (*in situ*).

Our study showed significant genetic differentiation among populations based on cpSSR and nSSR data, which resulted in geographically defined genetic clusters of populations. Four groups were detected in *L*. *auriculata*, which could be managed as four evolutionary units and assigned the highest priority protection. Considering the highest number of private alleles and haplotypes detected in the AY population, special attention should be paid to genetic resources from this region. To preserve all extant populations and their habitats, comprehensive *ex situ* conservation is viable by collecting seeds or seedling individuals from different populations from the three mountains, especially from the AY population.

Furthermore, artificial pollination is necessary to augment *in situ* populations because wild populations lack effective pollen^53^. Prohibition on deforestation in *L*. *auriculata* distribution areas should also be implemented. Taken together, these measures could protect *L*. *auriculata* from extinction.

## Conclusions

In summary, by using population genetics and ecological niche modeling analysis together, we were able to identify the many factors, historical and contemporary, have shaped population genetic structure in this endangered species. Moreover, geographical, environmental and climatic factors also contribute to patterns of genetic structure. Contemporary gene flow among *L*. *auriculata* populations showed drastic declines in comparison with historical gene flow, resulting in a high level of population divergence. *L*. *auriculata* populations underwent expansion after divergence and dramatically decreased to the current small size with relative population bottlenecks due to climate changes. Thus, both geographic environmental factors including climate changes have shaped patterns of genetic structure in *L*. *auriculata*, along with small population sizes, significant recent population bottlenecks, significant increases in the inbreeding coefficient and contemporary limited gene flow. Information on the genetic diversity, genetic structure, demographic history and integrated with ENM of *L*. *auriculata* can establish effective and efficient strategies for their conservation.

## Material and Methods

### Sample collection

*Litsea auriculata* occurs in deciduous broad-leaved forests or mixed coniferous broad-leaved forests at 500–1,250 m elevation, preferentially on valleys covered by deep and nutrient-rich soil, and their most important associated species are *Cyclocarya paliurus* (Juglandaceae), *Nyssa sinensis* (Nyssaceae), *Tilia tuan* Szyszyl. (Tiliaceae), and *Emmenopterys henryi* (Rubiaceae)^1^. *Litsea auriculata* is a diploid, dioecious and insect-pollinated tree (up to 25 m tall), with dark purple, one-seeded fruits (drupes) that are primary dispersed by birds^1,28,42,54^.

For this study samples were collected from their natural communities throughout east China including three provinces: Zhejiang, Anhui and Henan (Table 7). A total of 231 individuals of *L*. *auriculata* were collected from eight locations. Sample size ranged from 7 to 40 individuals per location (Table 7). Three populations (ZT, ZQ and ZD) are distributed in the Tianmu Mountains of Zhejiang Province; three populations (AS, AY and HX) were found in the Dabie Mountains of Anhui and Henan Province, and the last two populations (HY and HJ) occur in the Qinling Mountains of Henan Province (Fig. 1).

At least forty individuals within each location at an interval of at least 10 m were sampled. If the number of individuals in a particular site was less than 40, all available trees were then samples. Young leaves were randomly sampled from trees and immediately placed into plastic sealed bags with silica gel for fast drying. Subsequently these samples were stored at room temperature until use.

### DNA extractions and microsatellite analysis

Total genomic DNA from each of the samples was extracted from approximately 20 mg of silica-gel-dried leaves using a modified cetyltrimethyl ammonium bromide (CTAB) method^55^. After extraction, DNA were dissolved in 200 μL of sterilized water and kept at −30 °C until use.

For cpDNA analysis, we employed 33 universal chloroplast SSR (cpSSR) markers^56,57^; however, only seven of them (ccmp3, ccmp4, ccSSR-3, ccSSR-5, ccSSR-8, ccSSR-15 and ccSSR-22) showed polymorphism across all samples.

For nuclear DNA analysis, eight nSSR markers were polymorphic in *L*. *auriculata*. Three of these nuclear markers (Nese8, Nese9 and Nese10) were developed by^58^ and five of them (Litsea-10, Litsea-11, Litsea-36, Litsea-80 and Litsea-86) were obtained by Chiang, *et al*.^59^.

To amplify the SSR loci of *L*. *auriculata*, we used a tailed primer method to perform PCR: a U19 (5′-GGTTTTCCCAGTCACGACG-3′) was tailed to the 5′ end of the forward primer, and the U19 primer labelled with 6-FAM, VIC, NED or PET, was added to the PCR reaction mix. PCR amplification was performed in a reaction mixtures (10 μL) containing approximately 10 ng of template DNA, 0.2 mM of each dNTP, 1 × PCR buffer (16 mM (NH_4_)^2^SO_4_, 67 mM Tris–HCl (pH 8.8), 0.01% Tween-20), 1.35 mM of Mg^2+^, 0.5 U of BIOTaq DNA polymerase (Bioline, USA), and 0.5 mM of each of the three primers: a forward primer with a tail of the U19 sequence, a non-tailed reverse primer, and a 5′-labeled U19 primer.

The PCR reactions were conducted using a Veriti Thermal Cycler (Applied Biosystems). The PCR reaction was performed using the following cycling conditions: denaturation at 94 °C for 1 min; followed by 30 cycles of 30 s at 94 °C, 30 s at annealing temperature of primer pair and 1 min at 72 °C; and final extension at 72 °C for 5 min. The PCR products were genotyped on an ABI 3730 Genetic Analyzer (Applied Biosystems). Allele sizes were determined using the GeneMapper^TM^ analysis software version 4.0 (Applied Biosystems) according to a GeneScan^TM^ 500 LIZ^TM^ Size Standard (Applied Biosystems).

To investigate allele-size variation at the seven cpSSR loci, PCR products of each allele at each locus was subjected to DNA sequencing on an ABI 3730XL DNA Analyzer (Beijing Genomics Institute, Shenzhen, China).

### Genetic diversity and haplotype network construction

For cpSSRs, haplotype composition, number of haplotypes and number of unique haplotypes were calculated for each population. A unique haplotype was defined as one found in only that population. We constructed a haplotype frequency map to examine whether haplotype distribution was geographically structured. The haplotype diversity as defined by cpSSRs in each population was quantified in terms of the number of observed alleles per locus (*N*_a_), the effective number of alleles (*N*_E_), the Shannon index (*I*), diversity index, and unbiased diversity (*uh*) using GenAlEx 6.5^60^. Mean within-population genetic diversity, species total genetic diversity, and population genetic differentiation were calculated treating alleles as unordered (*h*_S_, *h*_T_ and *G*_ST_) and ordered (*v*_S_, *v*_T_ and *R*_ST_), following the methods described by Pons & Petit (1996) using PermutCpSSR 2.0^61^.

Phylogeographical structure was analysed by comparing two coefficients of population divergence (*G*_ST_ and *R*_ST_). *G*_ST_ is only based on haplotype frequencies, while *R*_ST_ also takes into account haplotype similarities. These two parameters were compared via a permutation test, using 10,000 permutations by PermutCpSSR 2.0^61^. A higher *R*_ST_ than the estimated *G*_ST_ indicates the presence of a phylogeographical structure. To examine the phylogenetic relationships of haplotypes, a haplotype tree was constructed in Network 5.0 using a median-joining haplotype network algorithm^62^. The distance between each pair of haplotypes was the sum of nucleotide differences between them over seven chloroplast microsatellite loci.

For nSSRs, genetic diversity was assessed at the species level, as gene diversity in the total population (*H*_T_) and average gene diversity within populations (*H*_S_)^63^, and at the population level, as the mean number of alleles per locus (*A*), allelic richness (*A*_R_), private alleles (*P*_A_), observed (*H*_O_) and expected (*H*_E_) heterozygosity. These calculations were performed using FSTAT version 2.9.3.2^64^ and GenAlEx 6.5^60^. Inbreeding coefficients (*F*_IS_) were calculated using FSTAT version 2.9.3.2^64^. The deviation of *F*_IS_ from zero was tested in each population using 1,000 permutation tests with a sequential Bonferroni correction^65^. The effective population sizes of each population (*N*_e_) were estimated at three levels of the lowest allele frequency (0.01, 0.02 and 0.05) with a 95% confidence interval using the program LDNe^66^. Significant tests of a linear parametric regression were performed for the two parameters (*A*_r_ and *H*_E_) against the latitude and longitude of the eight populations.

### Population genetic structure

Analysis of molecular variance [AMOVA, Excoffier, *et al*.^67^] of cpSSRs and nSSRs data was performed to estimate the total percentage variance attributable to difference within and among populations of *L*. *auriculata*. Analyses were performed using Arlequin version 3.5^68^, and significance tests were conducted using 10,000 permutations. Genetic differentiation among populations (*F*_ST(c)_ for cpSSRs, wright *F*_ST(n)_ for nSSRs) was also obtained using Arlequin version 3.5^68^.

For cpSSRs, Nei’s unbiased genetic distance for all population pairs [*G*_ST_, Nei^69^] was calculated using PopGene 1.32^70^. For nSSRs, pairwise *F*_ST_ between populations was calculated and tested for significant differences using FSTAT version 2.9.3.2 [1000 bootstrap permutation, Goudet^64^].

Correlations between the pairwise *G*_ST_ (Nei’s unbiased genetic differentiation, cpSSRs) or *F*_ST_/(1 − *F*_ST_) (nSSRs) values and the geographic distance matrix were evaluated by the Mantel test^71^ in GenAlEx 6.5 software with 9,999 random permutations^60,72^.

To determine the optimal number of clusters that compose the studied populations, we implemented the Bayesian algorithm analysis in STRUCTURE 2.3 software^73^. Fifteen independent runs were performed for each *K* between 1 to 8 without prior information using the admixture model and assuming correlated allele frequencies. Each run consisted of 1,000,000 MCMC (Markov Chain Monte Carlo) repetitions after burn-in with 100,000 iterations based on the LOCPRIOR model^74^, an admixture model and the correlated allele frequencies model (hereafter, the F-model) described by Falush, *et al*.^75^. The optimal *K* was determined using the Δ*K* method implemented in STRUCTURE HARVESTER^76^. CLUMPP version 1.1^77^ were used to summarize parameters across 15 runs at the optimal *K* value. DISTRUCT 1.1 software^78^ was used to visualize the STRUCTURE results after processing with CLUMPP.

### Approximate Bayesian computation (ABC)

To further understand the population demographic history of *L*. *auriculata*, we employed an approximate Bayesian computation (ABC) analysis in DIYABC v 2.1^79^ to test evolutionary scenarios based on nuclear loci. Populations covering the entire species distribution were analysed to infer the history of the genetic structure indicated by nSSR analysis. To simplify the scenarios in the ABC analysis, we pooled all populations into four large populations based on the results of STRUCTURE analysis of this core data set: Pop1 (ZT, ZQ and ZD population), Pop2 (AS and HX population), Pop3 (AY population) and Pop4 (HJ and HY population). Considering the result of haplotype diversity of *L*. *auriculata*, Pop3 (AY population) was inferred as the ancestry population. A set of ten evolutionary scenarios were built and tested (see Fig. 5): either a Simple split model (scenario 1), Hierarchical split model (scenarios 2–6), or Isolation with admixture model (admixture, scenarios 7–10). In these scenarios, t# represents time scale measured in number of generations and N# represents effective population size of the corresponding populations (Pop1, Pop2, Pop3 and Pop4). The range and distribution of priors for parameters used to describe these scenarios (effective population size, time of splitting or merging events, and rates of admixture in the case of merging events) are shown in Supplementary Table S3. For each scenario, 9 × 10^6^ simulated data sets were run and the most likely scenario was determined by comparing the posterior probabilities (with 95% confidence intervals) using the logistic regression method^80^. For each set of scenarios, the most likely scenario was the one with the highest posterior probability value. To check the goodness of fit of the scenario, principal component analysis (PCA) was performed on the first 100,000 simulated datasets of the reference table in the summary statistics using the “model checking” option in DIYABC. Finally, to assess confidence in the model choice, type I and type II error rates was estimated with 500 pseudo-observed datasets (PODs) using the option “evaluate confidence in scenario choice”.

### Detection of recently and historical migrants

To evaluate the direction and rates of recent migration (i.e., within the last several generations) among populations, the BAYESASS 3.0.4 program was used^81^. Preliminary runs were performed to adjust the MCMC mixing parameters of migration rates (*m*), allele frequencies (*a*) and inbreeding coefficients (*f*), to ensure proposed acceptance rates approximately 20–60%^81^. For the final runs, the mixing parameters *m* = 0.15, *a* = 0.4 and *f* = 0.5 were used. Ten independent replicates with different random starting seeds were performed with a burn-in of 5,000,000 iterations followed by 50,000,000 MCMC iterations and a sampling frequency of 2,000.

To investigate historical migration rates (*m*_h_) among populations, we used Migrate-N version 3.6 (http://popgen.sc.fsu.edu/Migrate/Migrate-n.html)^82^. MIGRATE is based on coalescent theory to estimate effective population sizes (Theta = θ, θ = 4 *N*eµ, where *N*e is historical effective population size) and symmetrical migration rates (*M*) [(*M* = *m*_h_/µ, µ is mutation rate per generation (10^−2^)] between population pairs. Migrate-N assumes that populations are in migration-drift equilibrium, that population sizes and migration rates are constant through time, and that populations are randomly sampled. We ran three replicates of Migrate-N using Microsatellite mode using a simulation of the single stepwise mutation model with constant mutation rates and starting parameters based on *F*_ST_ calculations. The program estimates the parameters θ and *M* using a Bayesian method^83,84^, both of which could be used to estimate the number of migrants per generation (*N*_m_) into each population using the equation 4 *N*_m_ = θ* *M*. We estimated θ and *M* with slice sampling and uniform prior distribution (for θ, range = 0–1000.0, delta = 100; for *M*, range = 0–2000.0, delta = 200). The three long chains were performed with 50,000-recorded genealogies at a sampling interval of 20 increments after discarding the first 10,000 genealogies (burn-in) for each chain. We used a static heating scheme at four temperatures (1.0, 1.5, 3.0, 1,000,000) to efficiently search the genealogy space. The confidence interval for θ and the migration parameter *M* was calculated using a percentile approach^84^.

### Bottleneck test

In a recently bottlenecked population, the observed heterozygosity is higher than the expected equilibrium heterozygosity estimated from the observed number of alleles^85^. To evaluate whether the population had undergone recent bottlenecks, we adopted Wilcoxon’s signed-rank test with 10,000 iterations using BOTTLENECK software version 1.2.02^86^. We ran BOTTLENECK using three possible mutation models: the Infinite Allele Model (IAM), the Stepwise Mutation Model (SMM) and the Two Phase Model (TPM; 30% IAM and 70% SMM).

### Ecological niche modelling

ENM were constructed using current climatic conditions and those of the LGM through the principle of maximum entropy model in Maxent version 3.4.1^87^. Maxent uses occurrence data along with environmental data to estimate the probability of species occurrence on the basis of a uniform probability distribution (maximum entropy) and on the presence data provided by the user^88^. *L*. *auriculata* occurrence points in the study were based on GPS data taken during our field work and from Nature Reserve records. We used all georeferenced localities. For occurrence points without geographic coordinates, which were then georeferenced by using the software Google Earth (http://www.ditu7.com/). A total of 26 occurrence points of *L*. *auriculata* were obtained to be used for ENM construction (Supplementary Table S4).

We used 19 bioclimatic variables with a resolution of 2.5 min (~5 km) from WorldClim database (http://www.worldclim.org/)^89^. These variables represent summaries of means and variation in temperature and precipitation, and characterize dimensions of climate considered particularly relevant in determining species distributions^89^.

We used random seed to test the ENM of *L*. *auriculata*. To obtain accurate prediction, we run the model making 100 replicates under the crossvalidation form of replication, 0.01 regularization multiplier, 10,000 background points, jackknife tests of variable importance and a maximum of 500 iterations. Finally, 75% of the localities were used to build the model and remaining 25% were randomly selected to test it.

In order to construct niche model for LGM (21,000 years ago- 21 kyr BP) conditions, we projected the current species’ bioclimatic niche onto past climate layers, which were downloaded from the WorldClim database at a resolution of 2.5 min as in the analysis above. Both the community climate system model (CCSM4)^90^ and model for interdisciplinary research on climate (MIROC-ESM)^91^ were used. Both models were run in Maxent using the settings chosen for current conditions. The area under receiver operating characteristic (ROC) curve (AUC) was then used to evaluate the model performance. An AUC score above 0.75 represent good model prediction^92^. All ENM predictions were then visualized in ArcGis 10.3 (http://www.arcgis.com/features/index.html), and the map of China (shapefile) is free downloaded from Global Administrative Areas version 2.8 (http://www.gadm.org/country).

## Electronic supplementary material

Supplementary Information
