# Supplementary material for: Understanding population structure and historical demography of Litsea auriculata (Lauraceae), an endangered species in east China
Source: Sci Rep. 2017 Dec 11;7:17343. doi: 10.1038/s41598-017-16917-x (PMC5725559; doi:10.1038/s41598-017-16917-x)
Supplement: Supplementary file 1 — Supplementary Information [file 41598_2017_16917_MOESM1_ESM.pdf]

Understanding population structure and historical demography of *Litsea auriculata* (Lauraceae), an endangered species in east China

Qifang Geng[1,2,5†], Lin Sun[1,2†], Peihua Zhang[1,2], Zhongsheng Wang[1,2*], Yingxiong Qiu[3], Hong Liu[4] and Chunlan Lian[5*]

[1]School of Life Sciences, Nanjing University, 163 Xianlin Road, Nanjing 210023, Jiangsu Province, China

[2]State Key Laboratory of Pharmaceutical Biotechnology, School of Life Sciences, Nanjing University, 163 Xianlin Road, Nanjing 210023, Jiangsu Province, China

[3] Key laboratory of Conservation Biology for Endangered Wildlife of the Ministry of Education, and college of Life Sciences, Zhejiang University, Hangzhou 310058, Zhejiang Province, China

[4]Department of Earth and Environment, International Center for Tropical Botany, Florida International University, Miami, FL 33199, USA

[5]Asian Natural Environmental Science Center, The University of Tokyo, 1-1-8 Midori-cho, Nishitokyo, Tokyo 188-0002, Japan

*Correspondence and requests for materials should be addressed to Z.-S.Wang (wangzs@nju.edu.cn) and C.-L. Lian (lian@anesc.u-tokyo.ac.jp)

†Q. F. Geng and L. Sun contributed equally to this work.

Supplementary Fig. S1 Mean values of the posterior probability (LnP(D)) from 15 runs for each value of K (1–8) and ΔK.

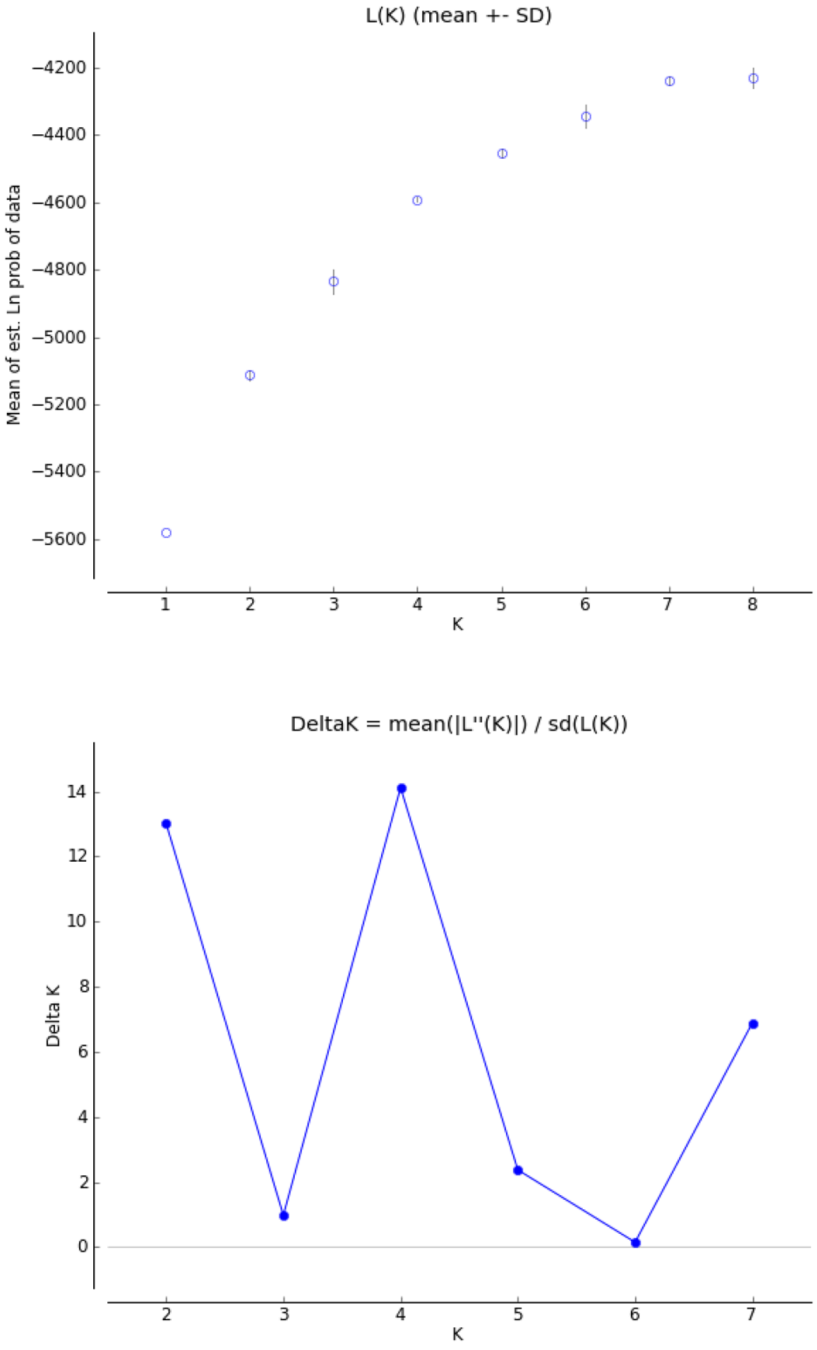

Supplementary Fig. S2 Logistic regression analysis obtained by DIYABC analysis.

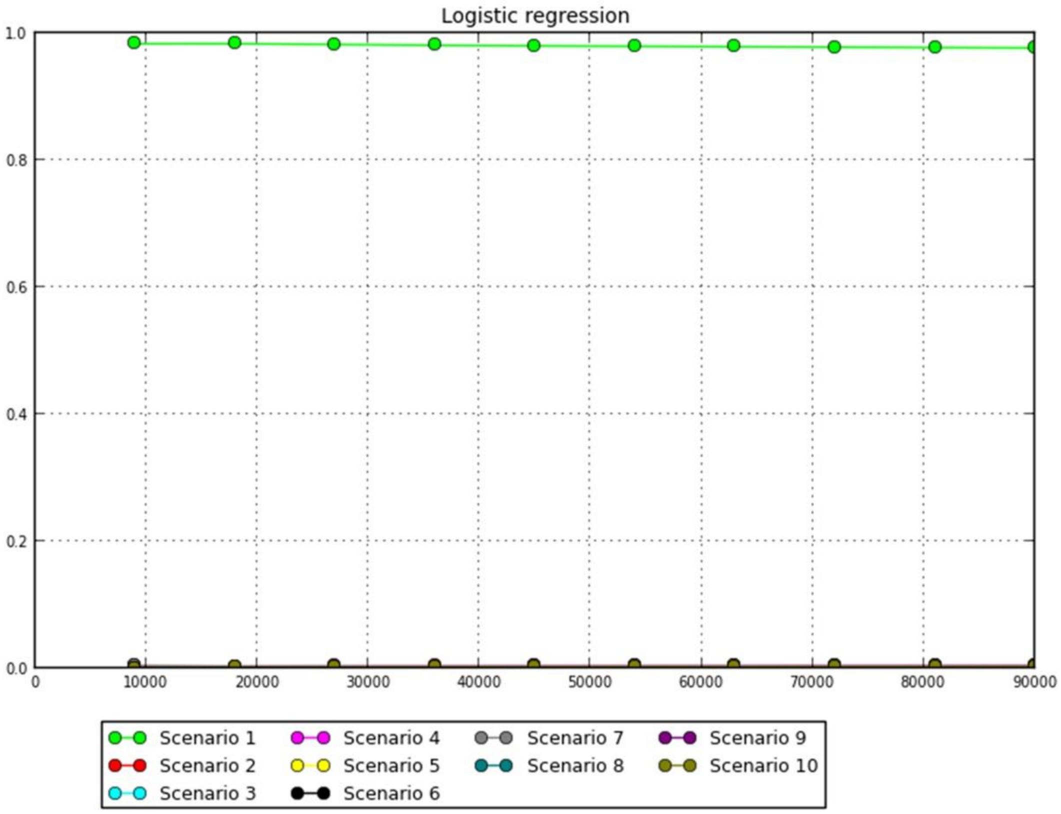

Supplementary Fig. S3 Principle Component Analysis (PCA) obtained by DIYABC analysis.

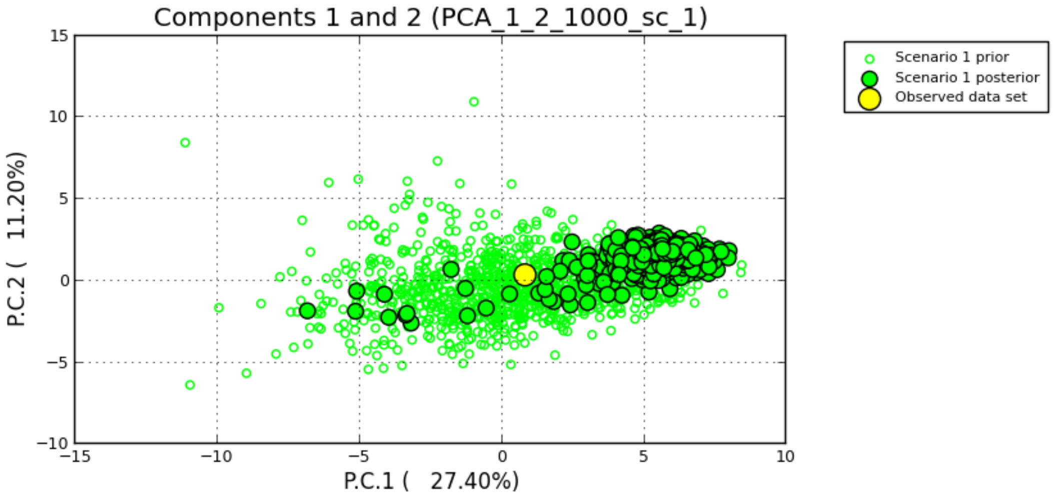

Supplementary Fig. S4 Prior and posterior distributions for each parameter obtained using DIYABC. Y–axis represents probability densities of prior and posteriors.

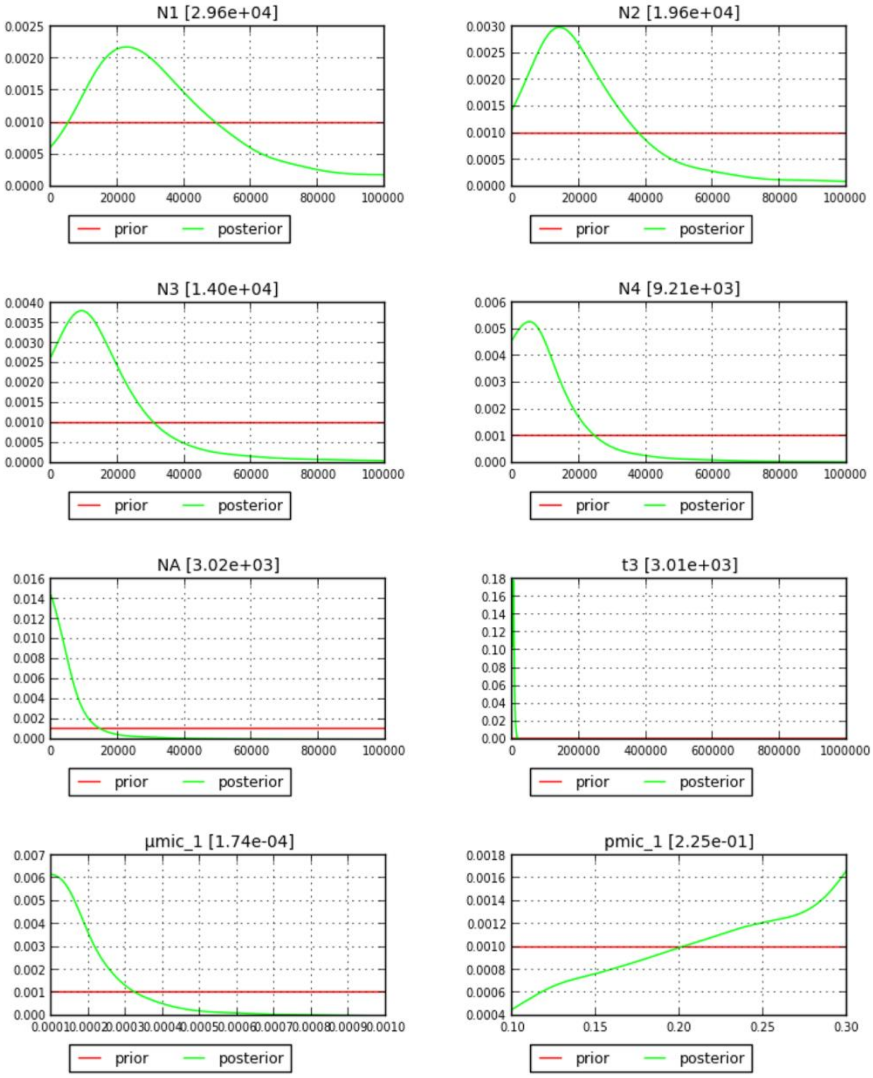

Supplementary Table S1 Comparison of the summary statistics for the observed data set and posterior simulated data sets.

Summary statistics	Observed value	*P*-value (simulated<observed)
Mean number of alleles in Pop1	7.8750	0.2614
Mean number of alleles in Pop2	7.0000	0.2283
Mean number of alleles in Pop3	5.5000	0.1237
Mean number of alleles in Pop4	5.1250	0.1221
Mean expected heterozygosity in Pop1	0.6515	0.2552
Mean expected heterozygosity in Pop2	0.5180	0.0697
Mean expected heterozygosity in Pop3	0.5122	0.0745
Mean expected heterozygosity in Pop4	0.4929	0.0653
F_{ST} (Pop1 and Pop2)	0.1687	0.9977**
F_{ST} (Pop1 and Pop3)	0.1588	0.9939**
F_{ST} (Pop1 and Pop4)	0.1418	0.9837*
F_{ST} (Pop2 and Pop3)	0.1718	0.9927**
F_{ST} (Pop2 and Pop4)	0.2760	0.9989**
F_{ST} (Pop3 and Pop4)	0.2865	0.9982**
Mean index of genotype likelihood (Pop1 and Pop2)	1.8557	0.7349
Mean index of genotype likelihood (Pop1 and Pop3)	1.9083	0.7127
Mean index of genotype likelihood (Pop1 and Pop4)	2.1084	0.7727
Mean index of genotype likelihood (Pop2 and Pop1)	1.7095	0.6485
Mean index of genotype likelihood (Pop2 and Pop3)	1.7111	0.5538
Mean index of genotype likelihood (Pop2 and Pop4)	1.9728	0.6796
Mean index of genotype likelihood (Pop3 and Pop1)	1.3801	0.3984
Mean index of genotype likelihood (Pop3 and Pop2)	1.5876	0.5390
Mean index of genotype likelihood (Pop3 and Pop4)	2.0127	0.7209
Mean index of genotype likelihood (Pop4 and Pop1)	1.6987	0.6496
Mean index of genotype likelihood (Pop4 and Pop2)	1.7383	0.6358
Mean index of genotype likelihood (Pop4 and Pop3)	2.3087	0.8493

*P <0.05, **P<0.01

Supplementary Table S2 Demographic parameters of scenario 1 obtained by DIYABC.

Parameter	Mean	Median	Mode	Quantile 2.5%	Quantile 5%	Quantile 95%	Quantile 97.5%
N1	34000	29600	17000	6610	8770	76400	86900
N2	24600	19600	11900	3930	5270	64000	78100
N3	18900	14000	7210	2720	3540	54700	70200
N4	14000	9210	5230	1720	2300	44000	61200
NA	6430	3020	378	127	251	24800	37900
t3	3740	3010	1730	519	740	8980	10900
Mean mutation rate_SSR	2.14E-04	1.74E-04	1.15E-04	1.05E-04	1.09E-04	4.50E-04	5.65E-04
Mean *P**	0.219	0.225	0.300	0.115	0.122	0.299	0.300

*parameter of the geometric distribution to generate multiple stepwise mutations.

Supplementary Table S3 Prior distributions of the parameters used in DIYABC analysis.

**Parameter**	Distribution	Min.	Max.
**Effective population size**			
N1, N2, N3,NA	uniform	10	100000
**Time of events (in generations backward in time)**			
ti (conditions: t1< t2, t2 <t3)	uniform	10	1000000
**Admixture rate (ra)**	uniform	0.001	0.999
**Microsatellites: Mutation model parameters**			
Mean mutation rate	uniform	0.0001	0.001
Mean coefficient p	uniform	0.1	0.3
Mean SNI rate	log-uniform	0	0

Supplementary Table S4 List of the occurrence points of *Litsea auriculata* used in the Species Distribution Modelling. The geographic coordinates are given in decimal units.

**species**	**Longitude**	**Latitude**
Litsea auriculata	119.4305	30.3399
Litsea auriculata	119.0015	30.0483
Litsea auriculata	118.9731	30.0369
Litsea auriculata	116.5488	31.0565
Litsea auriculata	116.0697	31.0315
Litsea auriculata	114.9999	31.8417
Litsea auriculata	112.0143	33.5357
Litsea auriculata	112.0007	33.5188
Litsea auriculata	119.2049	27.7497
Litsea auriculata	118.8985	30.1225
Litsea auriculata	121.0770	29.2630
Litsea auriculata	118.9589	29.7999
Litsea auriculata	116.1750	31.1240
Litsea auriculata	115.7705	31.1628
Litsea auriculata	118.8564	30.0906
Litsea auriculata	117.5347	30.0143
Litsea auriculata	118.7338	30.1367
Litsea auriculata	118.6456	30.5239
Litsea auriculata	116.5564	30.9891
Litsea auriculata	116.5061	31.0564
Litsea auriculata	117.6500	30.3167
Litsea auriculata	113.8333	32.5667
Litsea auriculata	113.5522	33.1721
Litsea auriculata	113.3575	33.1333
Litsea auriculata	111.8784	33.4403
Litsea auriculata	114.9609	28.9348
